# Critical Appraisal of Pancreaticogastrostomy After Pancreatoduodenectomy: Evolution, Evidence, and Future Prospects

**DOI:** 10.1002/jhbp.70072

**Published:** 2026-02-09

**Authors:** Samuel Menezes, Isabella R. Buonopane, Luís Felipe Leite, Anelise Poluboiarinov Cappellaro, Marcos Belotto

**Affiliations:** ^1^ Departament of Medical Sciences Universidade Federal da Bahia Salvador Brazil; ^2^ Department of Medical Sciences Universidade Federal Fluminense Niterói Brazil; ^3^ Centro Universitário Maurício de Nassau de Barreiras Barreiras Brazil; ^4^ Department of Surgery Hospital 9 de Julho São Paulo Brazil

## Abstract

Pancreaticogastrostomy (PG) emerged as a reconstructive option after pancreaticoduodenectomy (PD) due to its reported lower incidence of postoperative pancreatic fistula (POPF), but its superiority over pancreaticojejunostomy (PJ) remains unclear. We provide a narrative review that summarizes its evolution, technical variations, and comparative outcomes versus PJ. A narrative review from a comprehensive literature search was conducted using electronic databases of Medline/PubMed, EMBASE, and the Cochrane Library to identify relevant studies addressing surgical techniques, outcomes, and comparative analyses. PG offers anatomical, physiological advantages, including tension‐free anastomosis and gastric acid inactivation of pancreatic enzymes. Early RCTs showed similar clinically relevant POPF rates. Later trials supported PG for soft glands and small ducts, showing fewer intraperitoneal collections but more hemorrhage. Despite similar short‐term outcomes, long‐term randomized follow‐up shows worse exocrine function after PG than PJ, with higher fecal fat, lower fecal elastase‐1, and greater pancreatic atrophy. PG is an effective reconstruction option after PD, particularly in POPF high‐risk cases. However, it has higher bleeding rates and worse long‐term exocrine function than PJ. Reconstruction should prioritize pancreatic function, favoring PJ for most patients and reserving PG for selected soft, small‐duct, or high‐risk glands based on institutional expertise.

## Graphical abstract

Menezes and colleagues reviewed the evolution of pancreaticogastrostomy after pancreatoduodenectomy and its comparison with pancreaticojejunostomy. Randomized trials show similar rates of clinically relevant fistula, with potential advantages for pancreaticogastrostomy in selected high‐risk glands, but higher bleeding risk and worse long‐term exocrine function, supporting its selective use in appropriately chosen patients.

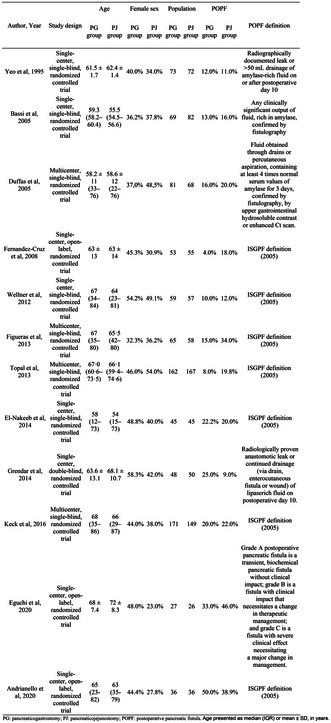

## 1 Introduction

Pancreaticoduodenectomy (PD) is a standard complex oncologic procedure for tumors of the pancreatic head and periampullary region. Until the 1970s, its use was limited due to high operative mortality rates of 25%–30% [1]. Although postoperative mortality has decreased to 2%–5%, PD for pancreatic ductal adenocarcinoma (PDA) still leads to 40%–50% morbidity, mainly from postoperative pancreatic fistula (POPF), prolonging hospital stays and raising treatment costs [2]. Reconstruction plays a pivotal role in determining outcomes, including length of hospital stay and hemorrhage [3].

Pancreaticojejunostomy (PJ) has been the standard reconstruction technique, consisting of an anastomosis between the pancreatic remnant and the jejunum to restore pancreatic drainage [4]. Despite refinements in technique, POPF rates remain high, ranging from 11% to 33% of the patients [5]. Therefore, pancreaticogastrostomy (PG) has increasingly gained attention over the past few decades due to its potential theoretical and technical advantages [6, 7, 8] This review provides a comprehensive analysis of the PG approach, outlining its historical evolution, assessing its current clinical applications, and discussing future perspectives.

## 2 Methods

**FIGURE 1:**
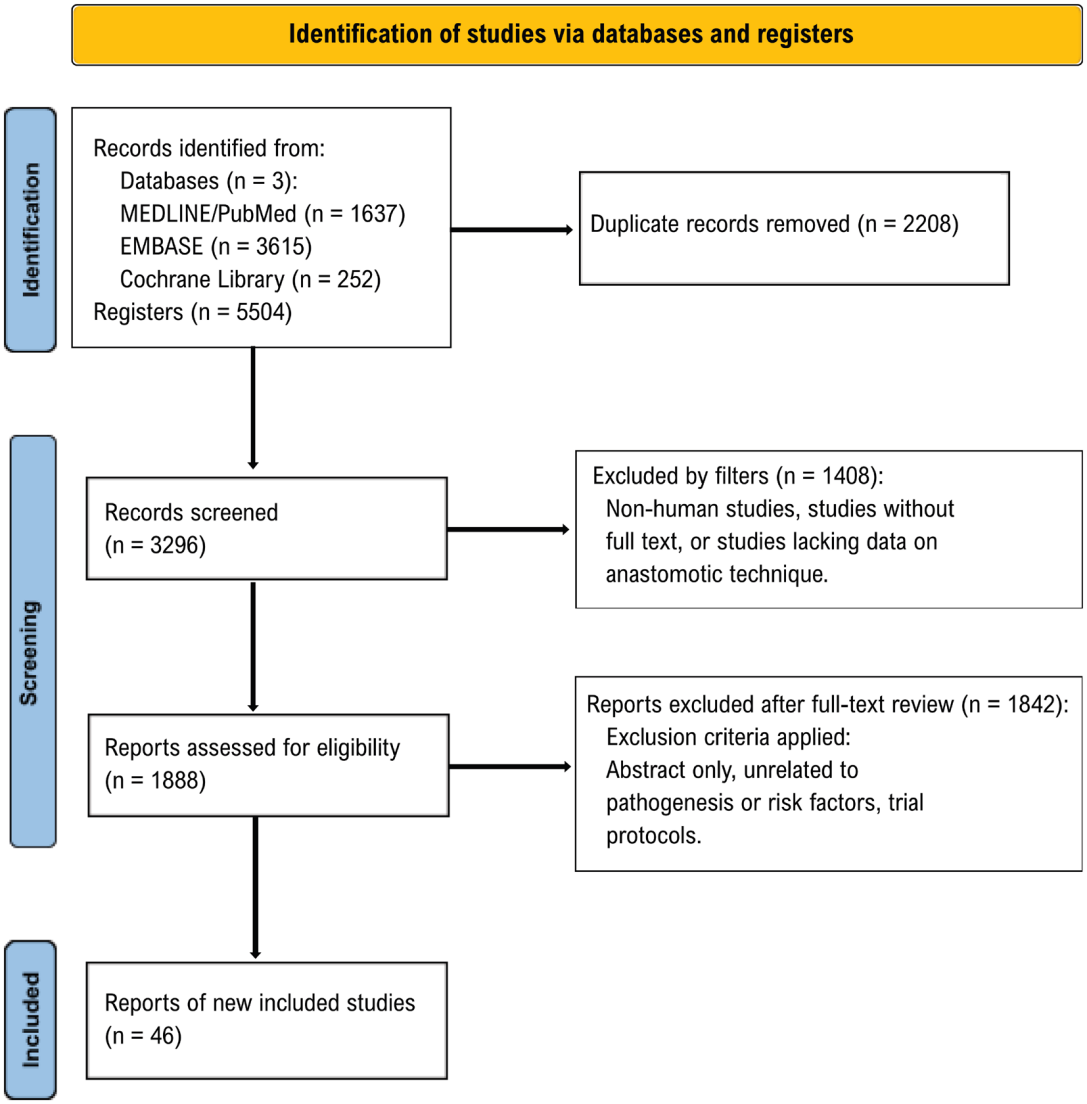
Flow chart of the search strategy employed.

This narrative review was conducted to critically appraise the evolution, techniques, and clinical outcomes associated with PG reconstruction following PD. A structured literature search was performed using PubMed, Embase, and the Cochrane Library, restricted to studies published in English and involving human subjects. Search terms included “pancreaticogastrostomy,” “pancreaticojejunostomy,” “pancreaticoduodenectomy,” “postoperative pancreatic fistula,” and “anastomotic reconstruction,” applied both independently and in combination to ensure comprehensive retrieval. Eligible studies included those addressing surgical techniques, risk stratification, technical innovations, and comparative outcomes between PG and PJ. Reference lists of key articles and recent meta‐analyses were hand‐searched to identify additional relevant literature. Editorials, conference abstracts, animal studies, case reports, and series with fewer than 10 patients were excluded. A PRISMA flowchart illustrating our study selection process is presented in Figure 1.

## 3 Historical Adoption of Techniques

Walter Kausch, in his 1912 report of the first successful PD (performed in Berlin in 1909), discussed the possible benefit of PG for reconstruction, but he described it as “unsafe and dangerous” [9]. In 1940, Allen Oldfather Whipple performed 37 consecutive PDs and introduced PJ as the reconstruction of choice, implanting the pancreatic duct into the jejunum to reconstruct the pancreatic leakage tract [10]. Although Waugh and Clagett first described PG in 1946, connecting the pancreatic remnant directly to the gastric wall, concerns over enzyme activation in the stomach and mechanical safety prevented its widespread adoption, leaving PJ the dominant technique for decades [11].

Beginning in the 1950s, scattered case reports rekindled interest in PG, and throughout the 1990s larger series demonstrated fistula rates comparable to PJ [12, 13, 14, 15, 16, 17, 18, 19, 20, 21, 22, 23, 24, 25, 26, 27, 28, 29]. These studies highlighted PG's anatomical advantages, yet PJ remained the standard. It was only after several randomized trials and, ultimately, the 2015 meta‐analysis by Hallet et al. (RR 0.91; 95% CI 0.65–1.28; *p* = 0.58) that robust evidence confirmed equivalent safety profiles for PG and PJ, cementing PG's resurgence as a validated alternative without displacing PJ as the default technique [30].

## 4 Theoretical and Technical Aspects of Pancreaticogastrostomy After Pancreaticoduodenectomy

PG leverages both anatomical proximity and the stomach's physiological environment to serve as a feasible reconstructive option alongside PJ following PD. Anatomically, the posterior gastric wall lies adjacent to the pancreatic remnant, allowing a tension‐free and well‐vascularized anastomosis on a thick, muscular substrate, particularly advantageous in soft glands with small ducts [6, 7, 31]. Physiologically, the acidic gastric lumen inactivates pancreatic enzymes, reducing autodigestive injury and the incidence of postoperative pancreatic fistula [32, 33].

PG reconstruction after PD can be fashioned by two principal techniques, each with distinct advantages. The duct‐to‐mucosa approach, though technically more demanding, ensures precise alignment of the pancreatic duct with the gastric mucosa and readily accommodates duct stenting, which may improve drainage and reduce fistula risk in patients with dilated ducts [34].

**FIGURE 2:**
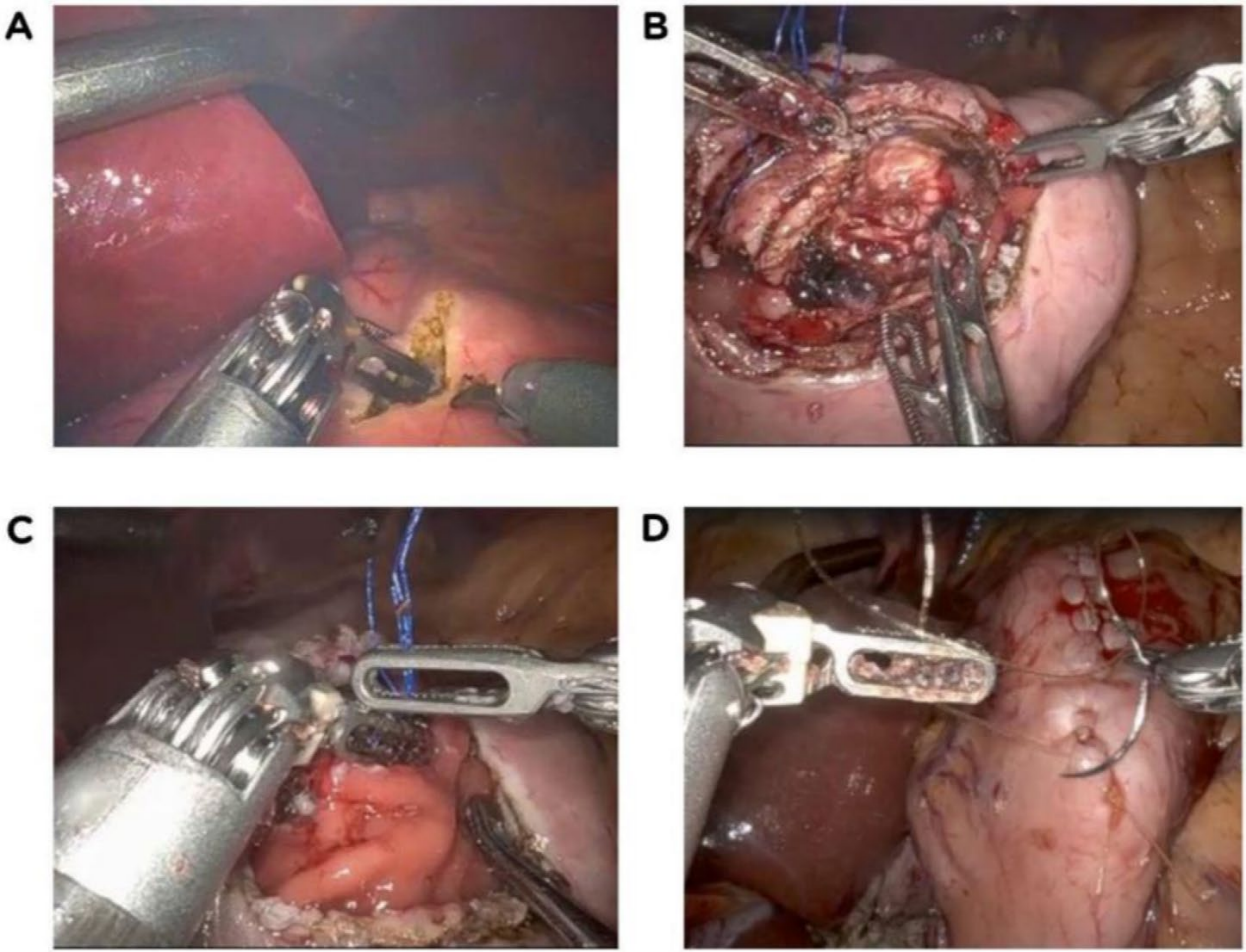
Stepwise pancreaticogastrostomy construction after pancreaticoduodenectomy. (A) Gastrostomy is created on the anterior wall of the stomach; (B) The pancreatic stump is gently invaginated into the gastric lumen; (C) The stump is secured in position using seromuscular or transfixing sutures; (D) Final aspect of the completed pancreaticogastrostomy.

By contrast, the invagination method is simpler to perform and particularly well suited to soft, friable glands: after creating a controlled gastrotomy in the posterior wall, the pancreatic stump is telescoped into the gastric lumen and secured to the gastric wall using seromuscular or transfixing sutures (Figure 2), resulting in a tension‐free anastomosis. Ultimately, duct‐to‐mucosa may be favored when duct size permits precise anastomosis and stenting, whereas invagination offers a rapid, reliable alternative in the setting of small ducts and soft gland texture.

Although invagination alone creates excellent tissue apposition, supplementing it with targeted adjuncts can further optimize anastomotic integrity and outflow. Internal or external stenting of the main pancreatic duct remains a cornerstone strategy to ensure unobstructed drainage and lowers intraductal pressure at the suture line [35, 36]. Beyond stents, the use of biodegradable meshes has shown promise in reducing clinically relevant POPF by mechanically buttressing the anastomosis and promoting tissue in‐growth [37]. In the randomized clinical trial by Jang et al., wrapping the pancreatic stump with a polyglycolic acid mesh plus fibrin glue significantly reduced the rate of clinically relevant postoperative pancreatic fistula (grade B/C) from 28.3% to 11.4% (*p* = 0.04), likely by enhancing both mechanical reinforcement and tissue integration [38]. Finally, vascularized seromuscular flaps, harvested from the gastric wall or omentum, provide an additional layer of reinforcement, improving seal strength and further mitigating leak risk [39].

## 5 Comparison of Pancreaticogastrostomy and Pancreatojejunostomy in Randomized Controlled Trials

In total, we analyzed 12 randomized controlled trials; the key studies and their main characteristics are summarized in Table 1. The first comparison in a Randomized Clinical Trial (RCT) of PG and PJ was performed by Yeo et al. at Johns Hopkins (1993–1995), who enrolled 145 PD patients to a two‐layer, stent‐free PG or PJ with routine closed‐suction drainage [40]. They observed nearly identical POPF rates (12.3% PG vs. 11.1% PJ; no significant difference), establishing that both reconstructions could be executed safely under standardized conditions.

**TABLE 1:** Summary of study design characteristics of the clinical trials discussed.

Author, Year	Study design	Age		Female sex		Population		POPF		POPF definition
		PG group	PJ group	PG group	PJ group	PG group	PJ group	PG group	PJ group	
Yeo et al., 1995	Single‐center, single‐blind, randomized controlled trial	61.5 ± 1.7	62.4 ± 1.4	40.0%	34.0%	73	72	12.0%	11.0%	Radiographically documented leak or > 50 mL drainage of amylase‐rich fluid on or after postoperative day 10
Bassi et al., 2005	Single‐center, single‐blind, randomized controlled trial	59.3 (58.2–60.4)	55.5 (54.5–56.6)	36.2%	37.8%	69	82	13.0%	16.0%	Any clinically significant output of fluid, rich in amylase, confirmed by fistulography
Duffas et al., 2005	Multicenter, single‐blind, randomized controlled trial	58.2 ± 11 (33–76)	58.6 ± 12 (22–76)	37.0%	48.5%	81	68	16.0%	20.0%	Fluid obtained through drains or percutaneous aspiration, containing at least 4 times normal serum values of amylase for 3 days, confirmed by fistulography, by upper gastrointestinal hydrosoluble contrast or enhanced Ct scan
Fernandez‐Cruz et al., 2008	Single‐center, open‐label, randomized controlled trial	63 ± 13	63 ± 14	45.3%	30.9%	53	55	4.0%	18.0%	ISGPF definition (2005)
Wellner et al., 2012	Single‐center, single‐blind, randomized controlled trial	67 (34–84)	64 (23–81)	54.2%	49.1%	59	57	10.0%	12.0%	ISGPF definition (2005)
Figueras et al., 2013	Multicenter, single‐blind, randomized controlled trial	67 (35–80)	65.5 (42–80)	32.3%	36.2%	65	58	15.0%	34.0%	ISGPF definition (2005)
Topal et al., 2013	Multicenter, single‐blind, randomized controlled trial	67.0 (60.6–73.5)	66.1 (59.4–74.6)	46.0%	54.0%	162	167	8.0%	19.8%	ISGPF definition (2005)
El‐Nakeeb et al., 2014	Single‐center, single‐blind, randomized controlled trial	58 (12–73)	54 (15–73)	48.8%	40.0%	45	45	22.2%	20.0%	ISGPF definition (2005)
Grendar et al., 2014	Single‐center, double‐blind, randomized controlled trial	63.6 ± 13.1	68.1 ± 10.7	58.3%	42.0%	48	50	25.0%	9.0%	Radiologically proven anastomotic leak or continued drainage (via drain, enterocutaneous fistula or wound) of lipaserich fluid on postoperative day 10
Keck et al., 2016	Multicenter, single‐blind, randomized controlled trial	68 (35–86)	66 (29–87)	44.0%	38.0%	171	149	20.0%	22.0%	ISGPF definition (2005)
Eguchi et al., 2020	Single‐center, open‐label, randomized controlled trial	68 ± 7.4	72 ± 8.3	48.0%	23.0%	27	26	33.0%	46.0%	Grade A postoperative pancreatic fistula is a transient, biochemical pancreatic fistula without clinical impact; grade B is a fistula with clinical impact that necessitates a change in therapeutic management; and grade C is a fistula with severe clinical effect necessitating a major change in management
Andrianello et al., 2020	Single‐center, open‐label, randomized controlled trial	65 (23–82)	63 (35–79)	44.4%	27.8%	36	36	50.0%	38.9%	ISGPF definition (2005)

*Note:* Age presented as median (IQR) or mean ± SD, in years.

Abbreviations: PG, pancreaticogastrostomy; PJ, pancreaticojejunostomy; POPF, postoperative pancreatic fistula.

Over the next decade, European trials refined this picture by exploring specific patient subgroups. In 2005, Bassi et al. reported that PG markedly reduced both overall and multiple‐event complication rates in high‐risk glands, defined by soft texture and small ducts, compared with PJ (25% vs. 69%; *p* = 0.002) [41]. While Duffas et al. [42] found no significant differences in POPF (16% vs. 20%) or overall morbidity (46% vs. 47%) between the two techniques, at the same year, Fernandez‐Cruz et al. [43] then applied the ISGPS definitions and demonstrated that gastric‐partitioned PG yielded significantly less clinically relevant fistula rates than PJ (4% vs. 18%; *p* < 0.01), reinforcing the value of tailoring anastomotic modifications to gland texture.

Larger, multicenter RCTs in the 2010s produced more nuanced findings. El‐Nakeeb et al. [44] compared Roux‐en‐Y PJ to PG in 120 high‐risk patients and found no difference in POPF, morbidity, or length of stay, suggesting that surgeon expertise can equalize outcomes. Topal et al. [45] stratified 329 patients by duct diameter and demonstrated a significant reduction in grade B/C POPF with PG (8.0% vs. 19.8%; OR 2.86; *p* = 0.002) and fewer severe complications. The RECOPANC trial (2015), involving 320 patients, similarly reported no overall difference in clinically relevant POPF or morbidity, though PG was linked to more low‐grade bleeding and hinted at a protective effect in low‐volume centers [46]. Grendar et al. [47] conducted a single‐center RCT enrolling 98 high‐risk PD patients to PG or PJ using standardized externalized stents and ISGPS POPF definitions. They observed POPF in 25% of the PG group versus 18% of the PJ group (*p* = 0.40). On multivariate analysis, soft pancreatic texture emerged as the only independent predictor of clinically relevant fistula (OR 5.89; 95% CI, 1.83–18.94; *p* = 0.003).

Most recently, high‐risk and functional‐endpoint trials have added further clarity. Andrianello et al. [48] enrolled only patients with a fistula risk score of 7–10, using externalized stents in both groups, found similar POPF rates (50.0% PG vs. 38.9% PJ; *p* = 0.48); however, a higher incidence of severe (Clavien–Dindo ≥ 3) complications was observed with PG, reaffirming PJ for the highest‐risk subgroup (0.25 [0.13] vs. 0.39 [0.17]; *p* = 0.04). Eguchi et al. [49] found comparable clinically relevant POPF rates (10% vs. 12%; p = 0.34) and a non‐significant trend toward less delayed gastric emptying with PG (11.1% vs. 23.1%; *p* = 0.42), underscoring PG's technical feasibility while highlighting the need for larger, blinded studies to confirm functional advantages.

Collectively, these RCTs underscore that neither PG nor PJ is universally superior across all clinical scenarios; however, no trial has demonstrated PJ to be superior to PG with respect to clinically relevant POPF, and when statistically significant differences have been observed, they have consistently favored PG in terms of fistula‐related outcomes.

These inconsistencies reinforce the importance of surgeon expertise and appropriate patient selection. A meta‐analysis by Fischer et al., which evaluated 31 studies on pancreatic surgery, showed that high surgeon volume was associated with reduced postoperative mortality (OR 0.29, 95% CI 0.22–0.37) and that high hospital volume reduced both mortality (OR 0.35, 95% CI 0.29–0.44) and major complications (OR 0.87, 95% CI 0.80–0.94) [50]. Similarly, Mehta et al., analyzing 2453 pancreatic resections, reported that high‐volume surgeons had lower 30‐day mortality (OR 0.54, 95% CI 0.33–0.87) and fewer complications (OR 0.71, 95% CI 0.55–0.93) [51]. Selection of patients may improve outcomes, as exemplified by Grendar et al., who demonstrated that soft pancreatic texture markedly increases the likelihood of clinically relevant POPF (OR 5.89, 95% CI 1.83–18.94, *p* = 0.003), and by Bassi et al., who in a cohort of 151 patients with soft glands at high risk for POPF showed that this is the subgroup in which PG achieved the greatest reduction in complications compared with PJ (69% to 25%, *p* = 0.002). Taken together, these data indicate that when surgeon expertise, operative execution, and perioperative management are consistently applied, the independent effect of reconstruction technique may diminish, contributing to the divergent results observed across trials.

Biologically, these clinical patterns are consistent with the anatomical and physiological features that may favor PG in selected contexts, including the short and well‐vascularized posterior gastric bed and the acidic gastric environment, which can attenuate enzymatic activity and limit autodigestive injury in soft glands with small ducts [52, 53, 54]. Within this framework, optimal reconstruction after pancreatoduodenectomy requires an individualized approach anchored in pancreatic texture, duct diameter, fistula risk score, and institutional expertise to guide the selection between PJ and PG and to mitigate postoperative morbidity.

## 6 Postoperative Morbidity Profile of Pancreaticogastrostomy

A meta‐analysis of 10 randomized controlled trials comprising 1629 patients, PG was associated with a significantly lower overall incidence of POPF compared to PJ (16.8% vs. 21.8%; OR 0.73; 95% CI 0.55–0.96; *p* = 0.02) [55]. Although this finding suggests a potential benefit in reducing pancreatic leakage, the subgroup analysis of clinically relevant fistulas (grade B/C) failed to demonstrate statistical significance (OR 0.61; 95% CI 0.34–1.09; *p* = 0.09), highlighting the need for cautious interpretation. Regarding gastric function, delayed gastric emptying occurred at similar rates between groups (21.7% vs. 20.0%; OR 1.06; 95% CI 0.67–1.69; *p* = 0.79), indicating no clear advantage of either technique in this domain. Conversely, PG was associated with a significantly lower incidence of intraperitoneal fluid collections (10.7% vs. 16.0%; OR 0.59; 95% CI 0.37–0.96; *p* = 0.03), which may reflect more efficient internal drainage and containment of enzymatic fluid.

However, these potential benefits must be balanced against a significantly higher rate of postoperative hemorrhage in the PG group (13.8% vs. 9.25%; OR 1.52; 95% CI 1.08–2.14; *p* = 0.02). The increased propensity for bleeding in PG is largely attributed to the highly vascularized nature of the gastric wall and the exposure of the pancreatic stump to acid and pepsin, which can erode the cut surface or the suture line [56].

A previous prospective cohort study of 274 patients found that while Grade A and B hemorrhage rates were similar, ISGPS Grade C PPH was significantly more frequent in the PG group (23.8% vs. 5.3% in PJ), associated with a higher rate of reoperation (28.6% vs. 8.4%). Despite this increased severity, the mortality rate for PPH‐related complications remained low and was not significantly different between groups [57]. Although PG is associated with higher rates of severe hemorrhage and reoperation in some series, the anatomical accessibility of the stomach facilitates rapid endoscopic intervention for ISGPS Grade B and intraluminal Grade C bleeding [58]. This unique therapeutic window allows for direct hemostasis, potentially downgrading what would be a surgical emergency (Grade C) to an endoscopic procedure. This capability effectively mitigates the failure‐to‐rescue rate, explaining why mortality remains comparable between the techniques despite the increased bleeding incidence.

Although short‐term results show similar exocrine function between PG and PJ, long‐term follow‐up revealed a significant divergence. In a 12‐year post‐trial reassessment of patients originally enrolled in Bassi et al. [41] trial, 34 individuals underwent structured morphological and functional pancreatic evaluation [59]. Despite similar BMI, endocrine function, quality of life, and symptom scores, patients who underwent PG exhibited significantly worse exocrine function: fecal fat excretion was higher (26.6 ± 4.1 vs. 18.2 ± 3.6 g/day), fecal elastase‐1 was lower (121.4 ± 6.7 vs. 170.2 ± 25.5 μg/g), and serum vitamin D was reduced (18.1 ± 1.8 vs. 23.2 ± 3.1 ng/mL). MRI also showed greater ductal dilation (4.6 ± 0.92 vs. 2.4 ± 0.18 mm) and reduced pancreatic volume (26 ± 3.1 vs. 36 ± 4.1 cm^3^) in the PG group, underscoring the impact of anastomotic drainage on gland atrophy. These observations, however, derive from a single‐center cohort with a modest sample size in which long‐term exocrine outcomes were secondary endpoints, and no randomized trial to date has been designed or powered primarily to assess long‐term exocrine or endocrine function after reconstruction.

In summary, PG modestly lowers the risk of POPF and intraperitoneal fluid collections, reflecting more contained enzymatic drainage, but incurs a higher rate of mainly low‐grade postoperative hemorrhage. Short‐term endocrine and exocrine outcomes are equivalent, yet the limited long‐term randomized follow‐up available one‐year follow‐up reveals greater exocrine insufficiency after PG, with increased fecal fat loss, reduced elastase‐1 levels, and imaging evidence of parenchymal atrophy. Thus, while PG remains a valid option for patients with soft glands or small ducts, its bleeding and long‐term exocrine risks demand that reconstruction choice be tailored to gland texture, duct size, and institutional expertise within a multidisciplinary, risk‐stratified framework. Looking forward, more definitive conclusions regarding long‐term pancreatic function will require prospective, preferably multicenter cohorts or extended RCT follow‐up using standardized functional metrics. Practical barriers to such studies include the need for prolonged follow‐up in a population with substantial competing oncologic mortality and pronounced heterogeneity in pancreatic enzyme replacement therapy, whereby pancreatic exocrine insufficiency is frequently underdiagnosed and PERT is inconsistently prescribed, dosed, and adhered to across centers and clinicians, directly affecting nutritional status, gastrointestinal symptoms, and biochemical markers of exocrine function [60].

## 7 Candidate Selection Criteria for Pancreaticogastrostomy

Surgeons choose the reconstruction method after PD based on intraoperative risk stratification using validated scoring systems for clinically relevant postoperative pancreatic fistula (CR‐POPF) [61]. The original Fistula Risk Score (FRS) proposed by the authors uses four key factors, pancreatic texture, duct size, pathology, and blood loss, to stratify patients by fistula risk during surgery [62].

Soft pancreatic texture, typically identified by gentle palpation of a friable gland, remains the strongest independent predictor of clinically relevant postoperative pancreatic fistula (CR‐POPF). In a meta‐analysis of the International Study Group of Pancreatic Surgery (ISGPS), including 66 studies and 25 599 patients, soft texture conferred a significantly increased risk of CR‐POPF compared with firm glands (OR 4.24; 95% CI 3.67–4.89; *p* < 0.01). A narrow main pancreatic duct (MPD) ≤ 3 mm further elevated risk, with an overall pooled OR of 3.14 (95% CI 2.53–3.90; *p* < 0.01), and up to OR 3.66 (95% CI 2.62–5.12; *p* < 0.01) in studies using ≤ 3 mm as a high‐risk cut‐off. Although individual studies using cutoffs < 4 mm or < 5 mm yielded variable results, the association remained significant when MPD was measured intraoperatively (OR 3.21; 95% CI 2.70–3.81; *p* < 0.01) [63]. New versions of the original Fistula Risk Score (o‐FRS) have been suggested to make risk assessment during surgery quicker and easier. Some versions leave out blood loss (a‐FRS), and others use only soft pancreas and a small duct (MPD ≤ 3 mm), like the ua‐FRS. Even with these changes, the original FRS is still the most commonly accepted method to predict risk [64].

Low‐risk patients (FRS 0–2), characterized by firm glands, wide ducts (> 3 mm), minimal blood loss, and non‐obese habitus, are well suited for PJ, benefiting from a straightforward duct‐to‐mucosa anastomosis and shorter operative times [65]. Intermediate‐risk cases (FRS 3–6) require nuanced judgment: PG may buffer enzymatic injury in soft glands, while PJ with duct stenting can secure drainage in moderate‐risk scenarios [62]. In high‐risk patients (FRS ≥ 7), where leak rates exceed 50%, PG leverages the stomach's thick, well‐blooded wall and acidic lumen to protect the anastomosis, though surgeons must remain vigilant for bleeding complications [65].

In relation to the existing evidence, this review builds directly on the two main quantitative syntheses that have shaped current practice. Earlier meta‐analyses, including the landmark synthesis by Hallet and the more recent 10‐trial, 1629‐patient meta‐analysis by Wang, pooled the classic randomized trials up to the RECOPANC study and concluded that pancreaticogastrostomy and pancreaticojejunostomy have broadly comparable short‐term safety when all patients are analyzed together [30, 55]. Our review extends these findings in three ways. First, we incorporate more recent randomized trials by Andrianello and Eguchi, which were not included in the 10‐trial meta‐analysis, thereby updating the comparative assessment of pancreaticogastrostomy versus pancreaticojejunostomy in very high‐risk glands and with regard to delayed gastric emptying. Second, we integrate long‐term follow‐up from the Bassi randomized cohort, showing greater exocrine insufficiency, ductal dilatation, and pancreatic atrophy after pancreaticogastrostomy despite similar early fistula rates, emphasizing that short‐term equivalence does not guarantee long‐term functional equivalence. Third, by framing the results of these trials around fundamental risk factors such as pancreatic texture and duct diameter, we move beyond a purely descriptive comparison and propose a concise, risk‐based framework in which pancreaticojejunostomy remains the default in firm, large‐duct glands, pancreaticogastrostomy is a selective option in soft, small‐duct or otherwise high‐risk anatomy, and reconstruction is ultimately tailored to institutional expertise and anticipated long‐term exocrine burden.

Emerging tools promise to refine this selection further [55, 66]. Preoperative CT‐based models estimate gland texture and duct size before the first incision, offering a “pre‐FRS” that aligns closely with intraoperative risk [66]. Incorporating patient age, ASA score, and nutritional markers like serum albumin into risk models may enhance accuracy and personalize reconstruction choices [67]. Refined risk models enable selection of PG or PJ tailored to each patient's profile, thereby optimizing outcomes in PD.

## 8 Conclusion

PG is an effective reconstruction option after PD, particularly in high‐risk cases. However, beyond its higher bleeding rates, current long‐term evidence indicates a clear exocrine disadvantage of PG compared with PJ, with potential implications for nutritional status and quality of life. Reconstruction strategy should therefore prioritize preservation of pancreatic function, reserving PG for carefully selected soft, small‐duct or otherwise high‐risk glands and tailoring the choice of anastomosis to gland texture, duct size, and institutional expertise.

## Funding

The authors have nothing to report.

## Conflicts of Interest

The authors declare no conflicts of interest.

## Data Availability

The data that support the findings of this study are available from the corresponding author upon reasonable request.
